# Emerging Role of Tet2 in the Regulation of Vascular Smooth Muscle Cell Identity During Arterial Calcification

**DOI:** 10.1016/j.jacbts.2025.101451

**Published:** 2025-12-17

**Authors:** Marie Piollet, Clément Cochain

**Affiliations:** a Université Paris Cité, PARCC, INSERM, Paris, France; b Institute of Experimental Biomedicine, University Hospital Würzburg, Würzburg, Germany

**Keywords:** arterial calcification, epigenetic, osteogenic transdifferentiation, vascular smooth muscle cell

**Figure:**
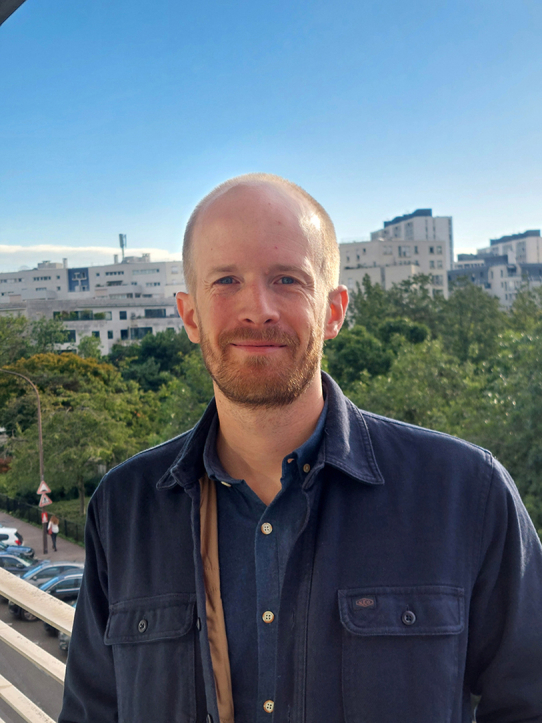

**Figure:**
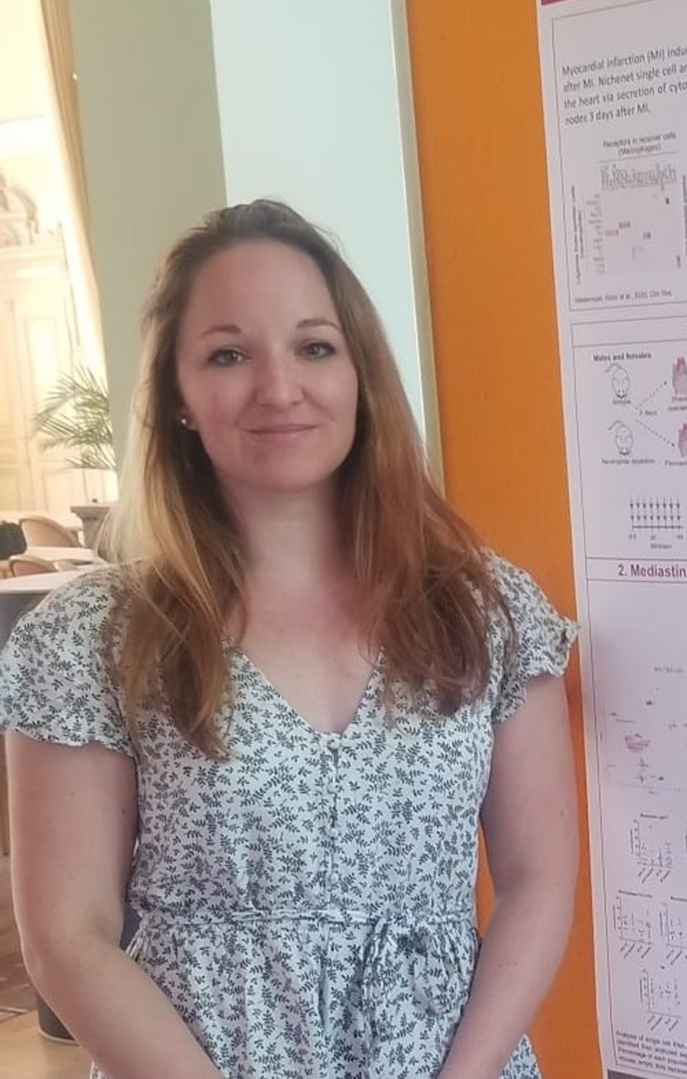

Vascular calcifications, defined as the deposition of calcium phosphate in the arterial wall, represent a characteristic pathological feature of vascular diseases during aging and in patients with cardiometabolic disorders, and are associated with clinical cardiovascular complications.^1^ Vascular calcifications develop via a mineralization process akin to osteogenesis, involving the interplay of inflammatory and stromal cells within the vessel wall, and culminating into the differentiation of highly plastic vascular smooth muscle cells (VSMCs) into an osteoblast-like, osteogenic phenotype.^1^ The epigenetic regulator Tet methylcytosine dioxygenase 2 (Tet2) has previously been associated with cardiovascular diseases (CVDs), as loss-of-function mutations in Tet2 drive clonal hematopoiesis of indeterminate potential (CHIP), a major CVD risk factor.^2^ However, beyond its involvement in CHIP, Tet2 was also proposed to be a regulator of VSMC plasticity, contributing to the maintenance of a homeostatic contractile phenotype,^3^ and recent studies have evidenced a functional role for Tet2 in VSMCs in vivo in experimental models of intimal thickening after arterial graft^4^ or vascular calcification.^5^^,^^6^

In this issue of *JACC: Basic to Translational Science*, Lee et al^7^ sought to investigate the involvement of Tet2 in VSMCs in vascular calcification, aiming to complement the growing mechanistic understanding of the role of Tet2 in vascular diseases, and especially in the control of VSMC plasticity in the context of medial aortic calcification.

Combining the use of a mouse model of medial aortic calcification induced by cholecalciferol (vitamin D_3_) overload and the analysis of human arterial samples from chronic kidney disease (CKD) patients, Lee et al^7^ first show a strong reduction of Tet2 mRNA expression and enzymatic activity in arteries with medial aortic calcification, both in their mouse model and in human samples. Using an inducible genetic mouse strain with VSMC-specific loss of Tet2 (*Myh11-CreER*^*T2*^*-Tet2*^*fl/fl*^), the authors provide evidence that Tet2 loss in VSMCs aggravates aortic calcification. In vitro, VSMCs showed increased osteogenic differentiation in response to ossifying medium, associated with loss or gain of permissive DNA and histone modifications in VSMC contractile (*Acta2*, *Myh11*) and osteogenic (*Runx2*, *Spp1*) gene promoters, respectively. In the cholecalciferol overload mouse model, mice with VSMC-specific Tet2 deficiency showed increased medial aortic calcification, with increased arterial stiffness and reduced arterial elasticity, 2 major vascular alterations associated to vascular calcifications. Lee et al^7^ furthermore identified an increase in VSMC apoptosis highlighting the importance of Tet2 in VSMC survival, increased infiltration of CD68^+^ macrophages, and increased expression of markers associated with a subset of Trem2^hi^ macrophages (*Spp1*, *Cd9*). Finally, activation of Tet2 following the administration of ascorbic acid protected against vascular calcification development by inhibiting VSMC osteogenic differentiation and apoptosis, an effect that was lost in mice with VSMC-specific Tet2 deficiency. The rescue effect of ascorbic acid on vascular calcification was confirmed ex vivo in human aortic tissue samples. Overall, the study by Lee et al^7^ provides convincing evidence of the involvement of Tet2 in inhibiting VSMC osteogenic differentiation and vascular calcification, putatively via the epigenetic control of VSMC identity. Given the importance of macrophage polarization in the development of vascular calcification,^8^ the authors’ data furthermore suggest a possible involvement of macrophages as drivers of arterial mineralization in this context.

However, this study presents limitations, and some mechanistic knowledge gaps remain to be investigated. Lee et al^7^ propose that loss of Tet2 in VSMCs selectively enrich the aorta with a subset of Trem2^hi^ macrophages. However, the methods used (immunofluorescence and bulk RNA-seq) do not allow concluding whether Tet2 loss only induces a global increase in macrophage recruitment or specifically affects gene expression programs in macrophages. A causal role of Trem2^hi^ macrophages in vascular calcification, potentially mediated by crosstalk with VSMCs, is also not demonstrated, and the accumulation of macrophages might merely be triggered by the increased VSMC apoptosis to facilitate their efferocytosis. Treatment with ascorbic acid represents a potentially interesting therapeutic strategy to enhance Tet2 activity and limits vascular calcification. However, in addition to its Tet2-activating properties, ascorbic acid is involved as an antioxidant or enzyme cofactor in many metabolic processes and in inflammation, all presenting a potential role in the development of vascular calcification.^9^ Finally, Lee et al^7^ used a mouse strain where the *Myh11-CreER*^*T2*^ transgene is born by the Y chromosome, precluding the inclusion of female mice, and the analysis of potentially relevant sex-specific effects.^10^

Interestingly, 2 other studies published in 2025 also highlighted a role of Tet2 as an inhibitor of VSMC osteogenic differentiation in vascular calcification and, using distinct models and approaches, gained complementary mechanistic insight. Fu et al^6^ showed that the metabolite alpha ketoglutarate attenuates vascular calcification, partially via the upregulation of Tet2 and the inhibition of the NLRP3 inflammasome signaling pathway. He et al^5^ demonstrated a protective role of Tet2 in preventing vascular calcification, proposing a mechanism independent of the DNA demethylation function of Tet2, where Tet2 facilitates the binding of HDAC1/2 to the promoter of *Runx2*, a known driver of VSMC osteogenic differentiation, thus inhibiting its transcription. These 3 recent studies evidence a role of Tet2 in vascular calcifications linked to its expression levels within the VSMC compartment, rather than directly related to loss-of-function mutations underlying increased CVD risk in CHIP. Although interferon-γ signaling has been proposed to repress Tet2 expression in VSMCs,^4^ the mechanisms mediating the downregulation of Tet2 expression within VSMCs in diseased arteries remain to be fully elucidated. Specific patterns of vascular calcification occur within arteries, with aortic valve calcification driving valve stenosis, intimal calcification being associated with atherosclerotic disease, and medial calcifications being prevalent in diabetic and CKD patients.^1^ The 3 aforementioned studies^5^, ^6^, ^7^ used models relevant to CKD and medial calcifications: whether Tet2 has a protective role in VSMCs across various types of vascular calcifications remains to be explored.

Altogether, these recent studies, including the report by Lee et al,^7^ converge towards an emerging protective role of VSMC-expressed Tet2 in limiting vascular calcifications, supporting the critical role of epigenetic modifiers in the control of VSMC identity, and placing Tet2 targeting as a promising strategy for the prevention and treatment of vascular calcification.

## Funding Support and Author Disclosures

The authors have reported that they have no relationships relevant to the contents of this paper to disclose.

## References

[1] Demer L.L., Tintut Y. (2008). Vascular calcification: pathobiology of a multifaceted disease. Circulation.

[2] Schuermans A., Honigberg M.C. (2025). Clonal haematopoiesis in cardiovascular disease: prognostic role and novel therapeutic target. Nat Rev Cardiol.

[3] Liu R., Jin Y., Tang W.H. (2013). Ten-eleven translocation-2 (TET2) is a master regulator of smooth muscle cell plasticity. Circulation.

[4] Ostriker A.C., Xie Y., Chakraborty R. (2021). TET2 protects against vascular smooth muscle cell apoptosis and intimal thickening in transplant vasculopathy. Circulation.

[5] He D., Ma J., Zhou Z. (2025). TET2 suppresses vascular calcification by forming an inhibitory complex with HDAC1/2 and SNIP1 independent of demethylation. J Clin Invest.

[6] Fu M., Lan Z., Ye Y. (2025). The metabolite alpha-ketoglutarate inhibits vascular calcification partially through modulation of the TET2/NLRP3 inflammasome signaling pathway. Kidney Int.

[7] Lee, Dunn (2026). JACC Basic Transl Sci.

[8] Li D., Fan C., Li X., Zhao L. (2024). The role of macrophage polarization in vascular calcification. Biochem Biophys Res Commun.

[9] Alberts A., Moldoveanu E.T., Niculescu A.G., Grumezescu A.M. (2025). Vitamin C: a comprehensive review of its role in health, disease prevention, and therapeutic potential. Molecules.

[10] Ward L.J., Laucyte-Cibulskiene A., Hernandez L. (2023). Coronary artery calcification and aortic valve calcification in patients with kidney failure: a sex-disaggregated study. Biol Sex Differ.

